# Naudin on Chronic Amygdalitis, &c.

**Published:** 1848

**Authors:** J. Naudin


### ARTICLE II.

### *On Chronic Amygdalitis and the treatment of Indurated Tonsils.* By Dr. J. NAUDIN.

The tonsils, by their situation, are often exposed to attacks of inflammation, which, after repeated occurrence, not unfrequently passes into a chronic state of induration. The disease is generally non-malignant, and affects both tonsils; carcinomatous induration being, on the contrary, much more rare, and affecting usually but one. The seat of this hypertrophy is neither the mucous membrane nor the cellular tissue, though their nutrition may also be altered, but in the glandular substance itself. The cause of the frequent occurrence of hypertrophy of glandular organs is that possessing a supply of arterial blood infinitely greater than is necessary for their nutrition, a large portion of which is destined to supply the material for secretion, any circumstance which produces a suppression of this secretion causes the excess of arterial bloood to become extended in the nutrition of the glandular substance, thereby inducing its hypertrophy and induration. Physicians are generally very neglectful of tonic inflammation of the tonsils, too often allowing the case to run on, and finally putting it into the hands of the surgeon for excision. The means, if any, employed with the view of reducing the tumors, are generally insufficient; and our author, instead of blisters, astringent gargles, iodine &c., substitutes gentle cauterization as employed in tonic inflammation of other organs. Instead of producing a slow progressive destruction of the tonsils, he aims at their preservation, and for this purpose employs a solution of nitrate of silver, three grains to one ounce of water, increasing the strength by three grains up to two ounces of the nitrate in the same quantity of water, and also applying the solid caustic to the surface of those hollows which usually exist in those tonsils, so that all parts may be equally affected. During one sitting the tonsils are painted twice or thrice ; the mouth is then well washed with water.

This cauterization must be repeated every two or three weeks until the tonsils are restored to their normal size, and then gradually discontinued; it produces no ill consequences, and even children speedily return to their play. Should the parts become accustomed to the caustic, it must either be discontinued for a time, or another substituted as Lugol's diluted solution of iodine. In two cases related by our author, nitrate alone was employed. Both, æt. 13 and 14, had been affected for years and were cured in two and a half to three months; in a third case, that of a girl, æt. 11, the disease ws extensive and obstinate, requiring four months' use of the caustic, besides the use of hyd. potass. and iodine internally and as ointment. In all these cases no return hss been observed after the lapse of years and the previous disposion to inflammation of the tonsils has been extingushed.—*Journ. de Toulouse* in Rank.'s Ab.

